# Draft genome sequence of *Acidiplasma cupricumulans* strain BH2^T^, isolated from a pregnant leachate solution of heap bioleaching

**DOI:** 10.1128/mra.00941-23

**Published:** 2023-12-08

**Authors:** Aleksandr G. Bulaev, Aleksandra V. Kanygina, Andrey V. Mardanov

**Affiliations:** 1 Winogradsky Institute of Microbiology, Research Center of Biotechnology of the Russian Academy of Sciences, Moscow, Russia; 2 Federal Research and Clinical Center of Physical-Chemical Medicine of Federal Medical Biological Agency, Moscow, Russia; 3 Institute of Bioengineering, Research Center of Biotechnology of the Russian Academy of Sciences, Moscow, Russia; Portland State University, Portland, Oregon, USA

**Keywords:** *Acidiplasma*, acidophiles, extremophiles, sulfide minerals

## Abstract

We report the draft genome sequence of a type strain *Acidiplasma cupricumulans* Strain BH2^T^, isolated from a pregnant leachate solution of industrial-scale chalcocite bioleach heap (Monywa, Myanmar). The genome is 1.7 Mbp long with a GC content of 34.2%.

## ANNOUNCEMENT

*Acidiplasma cupricumulans* BH^T^ (DSM 16651^T^, JCM 13668^T^), the type strain of the species *Acidiplasma cupricumulans*, is a facultatively aerobic, moderately thermophilic, extremely acidophilic, cell-wall lacked, pleomorphic archaeon oxidizing ferrous iron ions and utilizing organic nutrients that were isolated from acidic pregnant leachate solution of industrial-scale chalcocite bioleach heap (Monywa, Myanmar) (1). It was shown that archaea of the genus *Acidiplasma* often dominate in the technological processes of biooxidation of sulfide ores and concentrates as well as detected in a number of acidic environments in diverse geographical locations (1 – 6). Here, we report on the results of the assembly of the genomic sequence of *Acidiplasma cupricumulans* BH^T^ (DSM 16651^T^, JCM 13668^T^).

The strain was isolated as previously by Hawkes and co-authors as described in the work as representative of novel species *Ferroplasma cupricumulans* BH^T^ (1) and deposited in DSMZ-German Collection of Microorganisms and Cell Cultures (DSMZ) and Japan Collection of Microorganism (JSM) under accession numbers DSM 16651^T^ and JCM 13668^T^, respectively. Then, the strain was reclassified as representative of novel genus *Acidiplasma*, *Acidiplasma cupricumulans* (2). To perform the present study, the strain was provided by DSMZ collection. To obtain genomic DNA, the strain was grown as recommended by DSMZ collection at 55°С in a liquid medium containing ferrous iron and yeast extract (YE). To extract DNA, 0.5 L of the culture was grown. The cells were collected by centrifugation at 10,000 *g* using Allegra X22 centrifuge, and the biomass was washed twice with the acid solution containing the same component as the medium with the exception of ferrous sulfate and YE. Genomic DNA was extracted using PowerSoil DNA Isolation Kit (MO BIO, USA). Genomic library was prepared using TruSeq DNA preparation kit (Illumina, USA) according to the manufacturer’s recommendation. Sequencing was carried out using an Illumina HiSeq 1500 sequencer (Illumina, United States) producing 14,511,663 read pairs of 150 nucleotides (nt). Adapter sequences were removed using Cutadapt v1.8 software (7), and low-quality read ends were trimmed using Sickle v1.33 software (8). In total, 346,215,200 nucleotides were used for assembly, giving a coverage of 200. The reads were assembled *de novo* using SPAdes v. 3.6.0 software (9). Default parameters were used for all software. The assembly produced 312 contigs with a total genome size of 1,731,076 bp, an N50 value of 10,066 bp, L50 of 50, and a GC content of 34.2%. Annotation of the genome sequence performed using NCBI Prokaryotic Annotation Pipeline (10) revealed 1,813 coding sequences, respectively. Among these, 1,683 were identified as protein-coding sequences; 44 were identified as tRNA genes; and 83 were identified as pseudogenes.

## Data Availability

Sequence data associated with the annotated genome have been deposited in the NCBI BioProject accession number PRJNA295646. This whole-genome shotgun project has been deposited at DDBJ/ENA/GenBank under the accession number LKBH00000000. The version described in this paper is version LKBH00000000.1. The Illumina reads can be found under SRA accession number SRA—SRR25937293.
